# Use of Clomiphene Citrate in minimal stimulation *in vitro* fertilization negatively impacts endometrial thickness: an argument for a freeze-all approach

**DOI:** 10.5935/1518-0557.20180070

**Published:** 2018

**Authors:** Beverly G. Reed, John L. Wu, Laurice Bou Nemer, Bruce R Carr, Orhan Bukulmez

**Affiliations:** 1 Division of Reproductive Endocrinology & Infertility, Department of Obstetrics & Gynecology University of Texas Southwestern Medical Center. Dallas, Texas

**Keywords:** advanced reproductive age, clomiphene citrate, diminished ovarian reserve, endometrial stripe thickness, *in vitro* fertilization, minimal stimulation

## Abstract

**Objective:**

Minimal stimulation IVF is a treatment option that uses clomiphene citrate (CC). We sought to evaluate how CC impacts endometrial thickness during minimal stimulation IVF cycles.

**Methods:**

We retrospectively analyzed a cohort of 230 cycles in 119 poor ovarian response patients. The IVF cycles were studied in three groups: 130 minimal stimulation cycles, 29 mild stimulation cycles, and 30 conventional high dose gonadotropin releasing hormone (GnRH) antagonist cycles. Thirty-three minimal stimulation IVF patients had 41 frozen embryo transfers (FET) which allowed us to study whether the CC effects were prolonged.

**Results:**

Endometrial thickness in the minimal stimulation group was significantly lower than the mild and conventional stimulation groups (7.3±2.2mm versus 11.4±3.3mm versus 12.9±3.8mm, respectively, *p*<0.0001). In patients who underwent minimal stimulation IVF followed by FET, significantly thicker endometrial thickness was achieved during their FET cycles as compared to their minimal stimulation cycles (7.95±2.1mm versus 10.3±1.8mm, *p*<0.0001).

**Conclusion:**

We concluded that endometrial thickness is impacted during minimal stimulation IVF cycles. Since negative effects on endometrial thickness are not observed in the patients’ subsequent FET cycle, a freeze-all approach is justified to mitigate adverse endometrial effects of CC in minimal stimulation IVF cycles.

## INTRODUCTION

**Figure 1:**
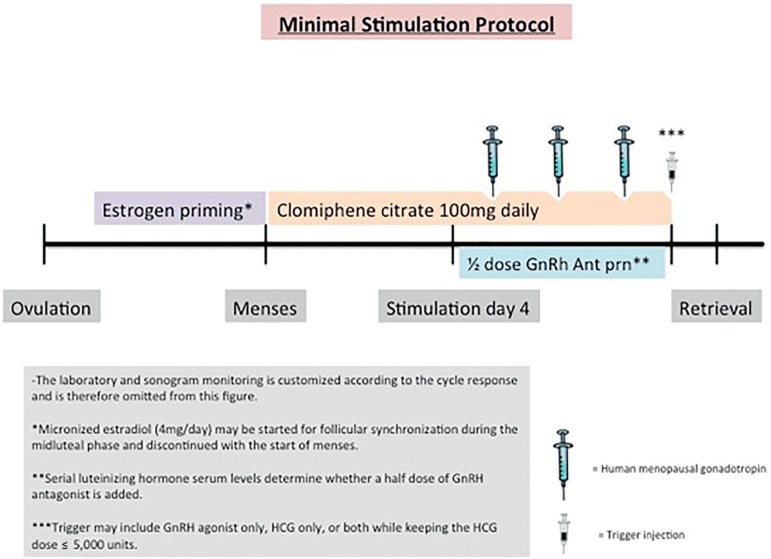
Minimal Stimulation IVF Protocol A visual representation of a commonly used minimal stimulation IVF protocol

Minimal stimulation *in vitro* fertilization (IVF) is an alternative to conventional high dose controlled ovarian stimulation (COS) for patients with diminished ovarian reserve or for expected poor responders. Advocates for minimal stimulation IVF note advantages that include reduced gonadotropin consumption and expense, reduction of ovarian hyperstimulation syndrome, and lower multiple pregnancy rates (Heijnen *et al*., 2007; Karimzadeh *et al*., 2010;Zhang, 2016; Zhang *et al*., 2016). In addition, the lower dose of gonadotropin used may allow for better quality oocytes and embryos (Baart *et al*., 2007; Haas *et al*., 2015). We offer the minimal stimulation approach to patients with expected poor ovarian response mainly due to diminished ovarian reserve and/or advanced reproductive age with a plan for embryo accumulation and subsequent frozen embryo transfer (FET) (Reed *et al*., 2015). A commonly prescribed minimal ovarian stimulation protocol uses daily clomiphene citrate (CC) and a small amount of gonadotropin on days 5, 7, and 9 of the ovarian stimulation (Figure 1) (Reed *et al*., 2015).

Clomiphene citrate (CC) is a selective estrogen receptor modulator. It binds to estrogen receptors in the hypothalamus which disrupts the normal estrogen feedback system in the body. In response to the CC perceived estrogen deficiency, the pituitary releases increased amounts of follicle stimulating hormone (FSH). The increased amounts of FSH allow for ovarian stimulation with lower doses of gonadotropin than would be traditionally used during an IVF cycle. The caveat is that CC may have some side effects that would be less than ideal during an IVF cycle. While CC can bind to all estrogen receptors, it can have agonistic effects in some tissues, while having antagonist effects in other tissues. One of the tissues is that is most rich with estrogen receptors is the endometrium. If CC binds to and blocks those receptors, the endometrial environment can be negatively impacted. There are no studies evaluating the effect of CC on the endometrium during IVF stimulation. However, there are studies that have been performed on intrauterine insemination (IUI) cycles that indicate CC may have a detrimental effect on the endometrial stripe (ES) thickness and pattern (Dehbashi *et al*., 2003; Haritha & Rajagopalan, 2003; Nakamura *et al*., 1997). Of note, most CC/IUI regimens only use CC for 5 days. During minimal stimulation IVF, CC is typically used for about 10 days or during the entire follicular phase of the treatment cycle until the day of trigger (Kawachiya *et al*., 2006). In addition, CC’s long half-life of about 5 days may allow for continued physiological effect even after the medication has been stopped (Ghobadi *et al*., 2009). Therefore, the negative impact of CC on the endometrium during IVF with prolonged CC use deserves further scrutiny.

Implantation and pregnancy rates are dependent on a number of factors. Endometrial thickness and pattern are two of the most important factors (Gingold et al., 2015; Noyes *et al*., 1995). A 2014 systematic review and meta-analysis of 22 studies found that an ES thickness of ≤ 7 mm resulted in a statistically significantly lower clinical pregnancy rate of 23.3% versus 48.1% (Kasius *et al*., 2014). Extrapolating from the studies involved with intrauterine insemination, we avoid fresh embryo transfer in patients who are undergoing minimal stimulation IVF despite the lack of data from IVF cycles to support this freeze-all practice. During our treatments, our observation has been that the endometrial lining is thinner than expected during minimal stimulation IVF. However, we have not observed endometrial thinning with all types of gentle stimulation. Mild stimulation IVF uses low doses of gonadotropin, but in contrast to minimal stimulation IVF, no CC is used. In the absence of CC use, we noticed the endometrial thickness seemed to be unaffected.

In order to confirm our anecdotal observations, we undertook this study to determine if the ES thickness is negatively impacted in minimal stimulation IVF as compared to other types of ovarian stimulation that do not utilize CC. Since minimal stimulation protocols are associated with lower estradiol levels, we anticipated that some may hypothesize that any differences seen in endometrial thickness may be due to that alone. Given this, we included a control group of patients who underwent a mild stimulation regimen that uses a low dose gonadotropin regimen without the use of CC.

Our solution to suspected endometrial thinning has been to freeze all of the embryos and later perform FET. Hence, an additional study aspect we sought to investigate was whether any potential endometrial changes seen during minimal stimulation IVF could be mitigated during a subsequent FET.

Since many of our patients undergo multiple cycles in order to be able to accumulate their desired number of frozen embryos, we also investigated whether the expected endometrial thickness changes worsen over these treatment cycles. CC is made up of two isomers (enclomiphene and zuclomiphene). Although enclomiphene is the more biologically active isomer, zuclomiphene has a longer half-life and has even been detected up to 456 hours after a single dose of CC (Ghobadi *et al*., 2009). Therefore, it may be logical to expect that endometrial thickness may become progressively thinner with multiple treatment cycles.

## MATERIALS AND METHODS

Our Institutional Review Board provided approval for retrospective data collection from January 2012-April 2016. Since the vast majority of our patients who undergo minimal stimulation IVF have diminished ovarian reserve and/or advanced reproductive age or are poor responders, we chose to use those characteristics as inclusion criteria to ensure that all compared groups included patients with a similar prognosis. We classified patients as poor responders if their age was ≥40 years old, if their ovarian reserve testing was abnormal (AMH <1.1 ng/ml))or total antral follicle count <7), or if a prior poor outcome occurred in a traditional IVF cycle (Ferraretti *et al*., 2011). Cancelled cycles before oocyte retrieval were excluded since we were unable to gather complete information on their ES.

The database was queried for patients with the inclusion criteria as noted above who underwent IVF. A total cohort of 230 controlled ovarian stimulation and FET cycles (119 patients) were included: 130 minimal ovarian stimulation cycles (75 patients), 29 mild stimulation cycles (16 patients) which utilized 150 IU daily dose of recombinant FSH starting either in the early follicular phase or luteal phase, 30 high dose gonadotropin-gonadotropin releasing hormone (GnRH) antagonist cycles (28 patients) which utilized 300 to 550 IU daily doses of gonadotropins. We also assessed 41 subsequent FET cycles of 33 minimal stimulation IVF patients.

### Stimulation Protocol Details

All IVF stimulation protocols were GnRH antagonist protocols. The use of oral contraceptive prior to the stimulation was avoided due to concern for ovarian suppression. Instead, estrogen priming was performed to allow for follicular synchronization prior to the stimulation. For minimal stimulation IVF (Figure 1), clomiphene citrate 100mg was given orally for 10 days starting on menstrual cycle day 2-3 (Reed *et al*., 2015).One hundred fifty international units (IU) of human menopausal gonadotropin (HMG) were added for days 5, 7, and 9. For mild stimulation, patients were on recombinant FSH 150 IU daily. For conventional IVF, the patients were started on high doses of recombinant FSH (usually 410 IU daily). Menopur^®^ 75 IU or 150 IU were usually added once the GnRH antagonist was started.

For all cycles, the start day for the antagonist varied based on follicle size and LH serum blood level. When the GnRH antagonist was started, it was usually only administered as a ½ dose per day. It was increased to a full dose of GnRH antagonist if the LH serum levels started to rise despite the ½ dose of GnRH antagonist. When the lead follicles were >=17mm, trigger with HCG (10,000 IU), leuprolide (2mg), or both (5,000 IU of HCG and leuprolide 2mg) occurred. Egg retrieval was performed 35 hours after trigger under IV sedation administered by an anesthesiologist. During the egg retrievals, flushing was performed. IVF was preferred and intracytoplasmic sperm injection (ICSI) was only used when indicated for reasons such as male factor or unexplained infertility.

Embryos were vitrified at the blastocyst stage. Vitrification of the embryos is performed per published methods (Kuwayama *et al*., 2005a; 2005b). Briefly, first Nunc four well dishes (Thermo Scientific, Grand Island, NY) are labeled; number 1 is designated as the left upper corner well and number 3 as the left lower corner. Equilibrium solution (ES) which includes dimethyl sulfoxide (DMSO) (Sigma- Aldrich, St Louis, MO), ethylene glycol (EG) (Sigma- Aldrich), synthetic serum substitute (SSS) (Sigma-Alrich), Quinn’s Advantage medium with HEPES (Origio, Male, Denmark) and vitrification solution (VS) with DMSO, EG, sucrose (Sigma-Aldrich), Ficoll (Sigma- Aldrich) and SSS were prepared. Then 1 ml of ES is added to wells 1 and 3 (left side of the dish) and 1 ml of VS is added to wells 2 and 4 (right side of the dish). The cryo dish is kept at room temperature shielded from light. Then the Cryotop (Kitazato, Tokyo, Japan) for each embryo is properly labeled. An embryo is removed from the culture media and transferred into ES media and kept there for about 10 minutes. Then the embryo is moved into the VS to be washed for about 50 seconds before beginning with the embryo loading into the cryotop under the microscope. The embryo is loaded onto the distal end of the cryotop’s tip and then the cryotop is quickly dropped directly into the liquid nitrogen.

### Embryo transfer protocols

Given the concern for endometrial changes, minimal stimulation IVF patients were not offered a fresh transfer. When those patients accumulated their desired number of embryos, they underwent frozen embryo transfer. Mild and conventional stimulation high dose IVF patients were eligible for transfer if their progesterone level was less than 1.5 and if their ES was greater than 7mm with an adequate pattern. If they did not meet these criteria, they would later undergo a frozen embryo transfer instead. Our frozen embryo transfer protocol consists of suppression with oral contraceptives and leuprolide acetate followed by an increasing estrogen cascade. Estrogen was usually given orally, but patches were occasionally used if the patient was not absorbing the oral estrogen well. Once the ES was > 7 mm with an acceptable pattern, progesterone in oil was administered intramuscularly and with blastocyst stage embryo transfer occurred on the 6^th^ day of progesterone. Methylprednisolone and doxycycline were given prophylactically at progesterone start, but they were stopped by the time of the transfer. Embryo transfer was performed under sonographic guidance with the embryo release 1.5-2cm from the top of the endometrial cavity. For fresh transfers, progesterone and estrogen were started day after the egg retrieval and transfer occurred on the 5^th^ day of progesterone.

Baseline demographics and labs were recorded including age during stimulation cycle, body mass index (BMI), and the last AMH level measured shortly before starting any stimulation. The baseline and peak endometrial thickness measurements were collected. Endometrial stripe thickness is measured in the mid sagittal plane. At our institution, the digital images were acquired by a reproductive endocrinology and infertility fellow and then approved by a reproductive endocrinology and infertility board certified faculty member. Baseline measurements were taken on or near the day of treatment start. During the stimulation, ultrasounds are performed after an increase in estradiol levels as early as the 4^th^ day of stimulation. The ultrasound monitoring frequency increases towards the end of the stimulation with the last ultrasound being the day of trigger for final oocyte maturation. Live birth rate per FET was also calculated.

The study characteristics are expressed as means ± standard deviation. Statistical analysis was performed using GraphPad Prism version 7.00 for Mac, GraphPad Software, La Jolla, California USA, www.graphpad.com. Homogeneity of data was evaluated for with Bartlett’s test. In factors where the homogeneity was verified (Age, BMI), one way ANOVA was used to compare the study groups. In the cases where Bartlett’s test demonstrated nonparametric data (ES, Peak estradiol, AMH, and Parity), the Kruskal-Wallis test was used instead of ANOVA to compare means. For ANOVA, post hoc analysis was performed using Tukey’s test. For the Kruskal-Wallis test, post hoc analysis was performed using Dunn’s test. Paired t-test and repeated measures ANOVA were used to compare the endometrial stripe thickness in the same patient during the minimal stimulation and her own subsequent FET cycle. A *p* value of ≤0.05 was considered to be statistically significant. Both per patient (first cycle) and per cycle analyses were performed.

A power analysis for the paired t test was calculated and we determined that 31 patients are needed to detect a medium effect size at a power of 0.80 and with an alpha level of 0.05. To see a small effect size, a sample size of 196 is required. A power analysis for repeated measures ANOVA (using 3 repeated measures) showed that a sample size of 12, 28, or 163 is required to see a large (*f*=0.4), medium (*f*=0.25), or small (*f*=0.1) effect size.

## RESULTS

There were no statistically significant differences in age, BMI, AMH level, or parity, indicating that the study groups included overall similar prognosis patients (Table 1).

**Table 1:** Baseline clinical information amongst study groups.

Clinical Factor	Minimal Stimulation (CC used) N = 130	High Dose Stimulation^1^ (CC not used) N = 30	Mild Stimulation (CC not used) N = 29	*p* Value
Age (y) at the time of the cycle	38.4±3.7	36.7±4.9	39.1±3.1	NS
BMI (kg/m^2^)	25.9±5	26±4.9	24.9±3.5	NS
AMH (pmol/L)	4.9±5.7	5.6±2.9	4.8±7.1	NS
Parity	0.3±0.6	0.4±0.9	0.4±0.5	NS

CC: Clomiphene citrate, BMI: Body mass index, AMH: Antimullerian hormone, NS: Not significant Values are the mean ± standard deviation. *p* value ≤0.05 was considered to be statistically significant. ^1^: High dose stimulation used was a GnRH Antagonist Protocol

**Figure 2:**
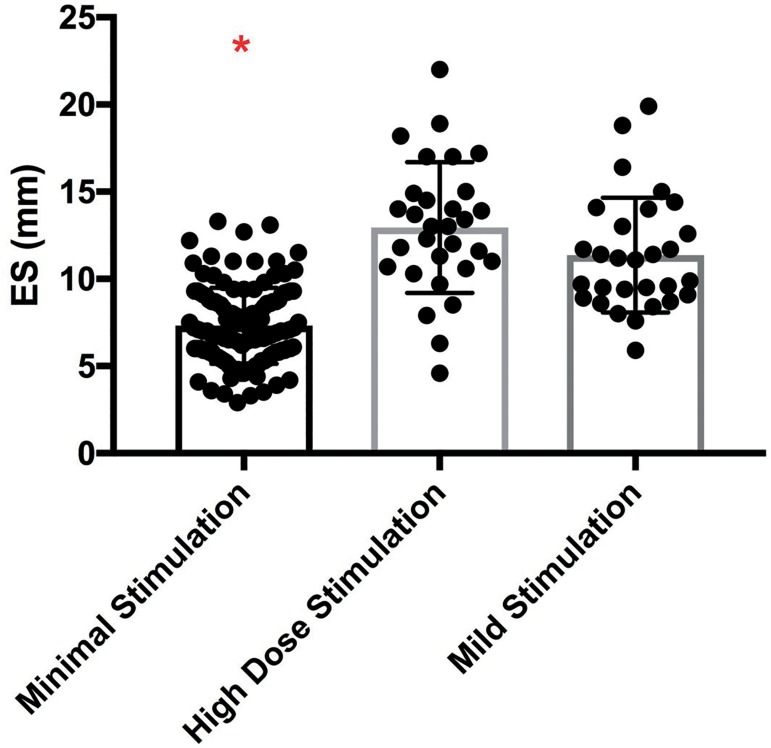
Comparison of endometrial thickness according to IVF protocol. Comparison of the peak endometrial stripe during minimal stimulation IVF, high dose GnRH antagonist, and mild stimulation IVF. Note: The posthoc analysis indicated that the high dose and mild stimulation groups were statistically significantly different from the minimal stimulation group as indicated by the red asterisk. * indicates *p*<0.01

The peak ES thickness per cycle in the minimal stimulation group was significantly lower than the high dose gonadotropin group or the mild stimulation group (7.3±2.2mm vs. 12.9±3.8mm vs. 11.4±3.3mm, respectively, *p*<0.0001, Figure 2). Interestingly, the standard deviations for each group differed resulting in nonparametric data. Graphically, we noted that the minimal stimulation IVF group had a narrower window of endometrial thickness amongst patients when compared to the wider ranges seen in the mild stimulation and traditional IVF study groups (Figure 2). The post hoc analysis indicated that there was no difference in ES between the high dose gonadotropin group and the mild group and it confirmed that both of the former had a statistically significant difference in ES when compared to the minimal stimulation group’s ES measurements.

To ensure that the patients who had multiple cycles did not skew the overall results, we also performed a per patient analysis. For the per patient analysis, only the patient’s first cycle was analyzed. This analysis including 119 patients confirmed the same findings. The mean peak ES thickness was 7.5±2.4mm vs. 12.9±3.9mm vs. 11±3.4mm, in the minimal stimulation, high dose gonadotropin stimulation, and the mild stimulation protocols, respectively (*p*<0.0001). Hence, the first cycle analysis did not change our conclusions.

**Figure 3:**
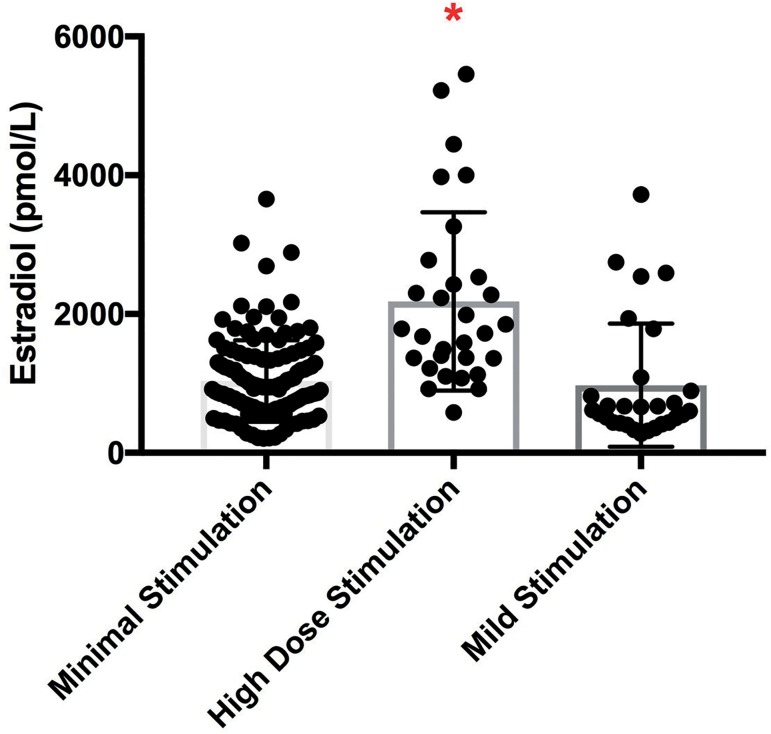
Comparison of peak serum estradiol levels according to IVF protocol. Comparison of the peak estradiol level in pg/mL during minimal stimulation IVF, high dose GnRH antagonist, and mild stimulation IVF. Note: The posthoc analysis indicated that the minimal and mild stimulation groups were statistically significantly different from the high dose GnRH antagonist group as indicated by the red asterisk. * indicates *p*<0.01

The mean peak estradiol levels measured on the day of trigger were significantly higher in the high dose gonadotropin group as compared to both the minimal or mild stimulation groups (Figure 3).

**Figure 4:**
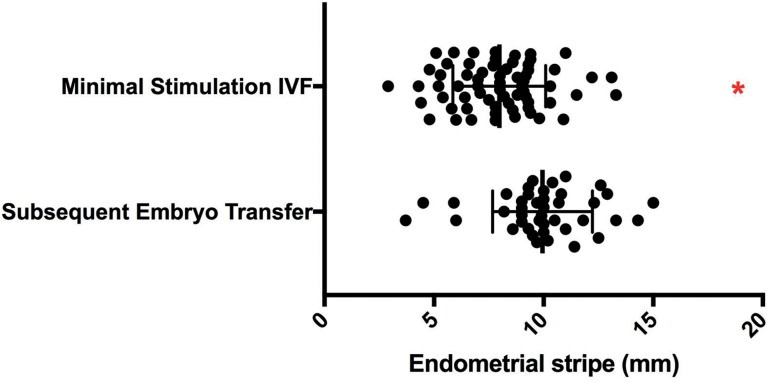
Matched comparison of endometrial thickness between minimal stimulation IVF and the patient’s subsequent frozen embryo transfer peak endometrial thickness during minimal stimulation compared to the patient’s subsequent frozen embryo transfer (FET) cycle peak endometrial thickness * indicates *p*<0.01

**Figure 5:**
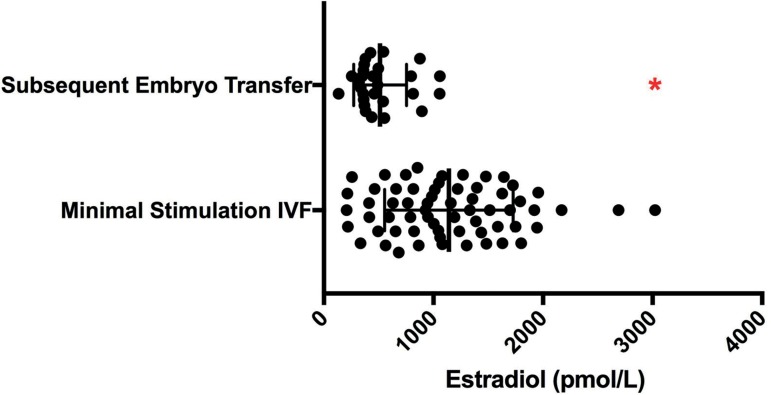
Matched comparison of peak serum estradiol level between minimal stimulation IVF and the patient’s subsequent frozen embryo transfer peak estradiol level during minimal stimulation IVF compared to the patient’s subsequent FET cycle * indicates *p*<0.01

Thirty-three patients had both their minimal stimulation IVF cycle(s) and their subsequent FET cycle(s) available for review. Significantly thicker endometrial stripes were achieved during their subsequent FET cycles (10.3±1.8mm vs. 7.95±2.1mm, *p*<0.0001, Figure 4). Peak estradiol levels are shown in Figure 5. When compared to the matched minimal stimulation cycle, the corresponding FET cycle had a statistically significantly lower mean peak estradiol, once again indicating that the estradiol level is not the key factor in endometrial thickness differences in these study groups. The live birth rate per FET in these patients was 41% (17/41 patients).

**Figure 6:**
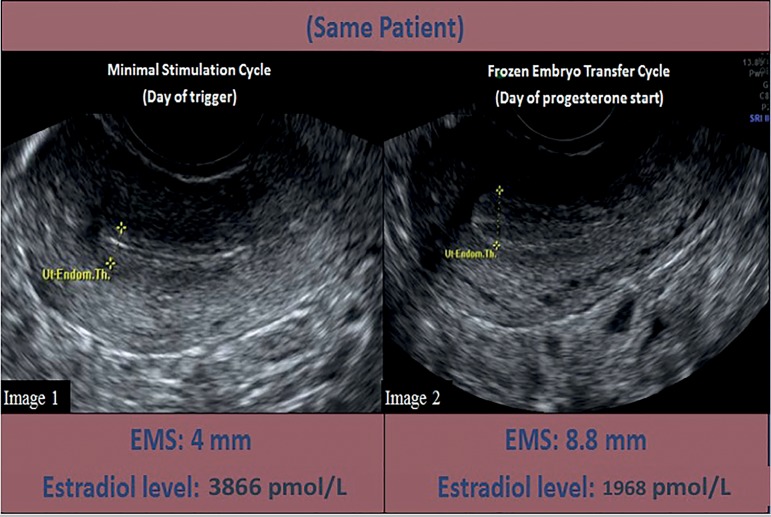
Endometrial thickness comparison in the same patient who underwent minimal stimulation IVF and frozen embryo transfer **Image 1**: Sonographic image of a patient’s endometrial lining on the trigger day of her minimal stimulation cycle. Her endometrial lining measures 4 mm and her estradiol level is 3866 pmol/L. **Image 2**: Sonographic image of the same patient’s endometrial lining on the day of progesterone start during her subsequent frozen embryo transfer cycle. Her endometrial lining measures 8.8 mm and has a trilaminar appearance. Her estradiol level is 1968 pmol/L.

We have included representative images of a patient’s endometrial thickness and pattern on the day of trigger during her minimal stimulation cycle compared to her endometrium on day of progesterone start during her subsequent FET (Figure 6).

There were 38 patients who underwent 2 or more consecutive minimal stimulation IVF cycles. A paired t test was performed to compare their endometrial thickness between their first and second cycle and no significant difference was found (7.4±1.8mm vs. 7.1±1.9mm, *p*=0.3). Our power analysis indicates that our sample size allows us to reasonably rule out large or medium changes. However, our sample size was not sufficient for this portion of the study to rule out smaller changes. There were 14 patients that underwent 3 consecutive minimal stimulation IVF cycles. Once again, there were no differences found for the mean peak endometrial thickness for their first, second, and third cycles (7.7±1.7mm vs. 7.0 ±1.8mm vs. 7.7±2.1mm, *p*=0.44, by repeated measures ANOVA). The power analysis for this part of the study only allowed us to rule out large changes. Medium or smaller changes to the endometrium cannot be ruled out with the small sample size. In this patient population, no significant differences were noted in the ES thickness measurements over repetitive minimal stimulation cycles, indicating that the CC thinning effect does not accumulate or worsen over time. However, further data collection will be continued in the future to rule out smaller changes not detected in this study.

## DISCUSSION

As infertility care is shifting towards an alternative lower dose approach, we must continuously evaluate how changing one part of a stimulation cycle can affect other aspects of the cycle. Here, we have shown that the use of CC during minimal stimulation IVF negatively impacts endometrial thickness. With the availability of vitrification, freezing all of the embryos in stimulation cycles that use CC seems to be a prudent approach while we further investigate mechanisms behind this practice. Knowing that the endometrium is affected during minimal stimulation IVF is also important when interpreting past minimal stimulation studies where fresh embryo transfer was utilized (Zhang *et al*., 2010).

Our study has some strengths that make it novel and that help us to better understand the physiology in assisted reproduction technology (ART) cycles. First, one might suppose that a thinner endometrium might be secondary to the lower estradiol levels that are observed during a low dose stimulation. However, by inclusion of a mild stimulation regimen that does not use CC, we showed that endometrial thickness similar to that seen in a high dose GnRH antagonist IVF cycles is easily achieved, despite having lower estradiol levels when compared to high dose stimulation. Inclusion of the mild stimulation group strengthens the argument that the endometrial thickness changes are likely due to CC rather than the lower estradiol levels.

An additional interesting aspect to this study was the consideration of the minimal stimulation patients’ endometrial thickness during their subsequent FET. The significant increase in endometrial thickness seen in their subsequent FET demonstrates that these patients certainly had the potential to develop a higher endometrial thickness, even with lower peak estradiol levels than those in their minimal stimulation cycles, but that there was a factor limiting it during the stimulation. In addition, we found that there is no additive effect with multiple minimal stimulation IVF cycles and that the mean peak endometrial thickness stays similar amongst repetitive cycles.

We acknowledge some limitations in our study. First, we chose to examine endometrial thickness given the objectivity of numerical data. However, we acknowledge that above a certain threshold of endometrial thickness, the endometrial pattern at the time of embryo transfer may be just as critical (Gingold *et al*., 2015). Second, our study was retrospective, single center, and has overall small numbers. Despite the small numbers, we were able to show a difference. In the portion of the study where no difference was seen (comparing ES in back to back minimal stimulation cycles), we acknowledge that there may have been small differences not seen due to low sample size. While this limits the study, we feel that the overall findings are logical and biologically plausible. Therefore, based on our data, a freeze-all approach should be considered in minimal stimulation IVF cycles which involve continuous daily CC use. We have current ongoing investigations based on this pilot study that will evaluate the histological, molecular changes, and genomic changes that occur with the use of prolonged CC in minimal stimulation cycles as compared to high dose GnRH antagonist ovarian stimulation for IVF.

While we feel that our study guides us towards freeze all, we realize that some may not feel the same. For those who are currently performing minimal stimulation IVF with the use of fresh transfer, there may also be concerns regarding the additional use of resources for a FET cycle when compared to a fresh transfer. Fortunately, we are able to offer a pricing structure at our institution that allows our patients to undergo minimal stimulation IVF and FET at a much more affordable price when compared to traditional IVF with a fresh transfer. We strongly believe that the endometrial thickness is affected by prolonged CC use, but we are curious as to whether it is the stromal layer or glandular layer (or both layers) that are affected. Our clinical findings from this study have led us to our next project in which we are studying the endometrial microscopic and genomic data to further understand the mechanistic functions by which CC affects the endometrium.
